# Neutralizing Antibody Responses to Antigenically Drifted Influenza A(H3N2) Viruses among Children and Adolescents following 2014-2015 Inactivated and Live Attenuated Influenza Vaccination

**DOI:** 10.1128/CVI.00297-16

**Published:** 2016-10-04

**Authors:** Min Z. Levine, Judith M. Martin, F. Liaini Gross, Stacie Jefferson, Kelly Stefano Cole, Crystal Ann Archibald, Mary Patricia Nowalk, Michael Susick, Krissy Moehling, Sarah Spencer, Jessie R. Chung, Brendan Flannery, Richard K. Zimmerman

**Affiliations:** a Influenza Division, Centers for Disease Control and Prevention, Atlanta, Georgia, USA; b Department of Pediatrics, University of Pittsburgh, School of Medicine, Pittsburgh, Pennsylvania, USA; c Department of Immunology, University of Pittsburgh, School of Medicine, Pittsburgh, Pennsylvania, USA; d Center for Vaccine Research, University of Pittsburgh, School of Medicine, Pittsburgh, Pennsylvania, USA; e Department of Family Medicine, University of Pittsburgh, School of Medicine, Pittsburgh, Pennsylvania, USA; f Graduate School of Public Health, University of Pittsburgh, Pittsburgh, Pennsylvania, USA; g Atlanta Research and Education Foundation, Atlanta, Georgia, USA; h Battelle, Atlanta, Georgia, USA; Vanderbilt University Medical Center

## Abstract

Human influenza A(H3N2) viruses that predominated during the moderately severe 2014-2015 influenza season differed antigenically from the vaccine component, resulting in reduced vaccine effectiveness (VE). To examine antibody responses to 2014-2015 inactivated influenza vaccine (IIV) and live-attenuated influenza vaccine (LAIV) among children and adolescents, we collected sera before and after vaccination from 150 children aged 3 to 17 years enrolled at health care facilities. Hemagglutination inhibition (HI) assays were used to assess the antibody responses to vaccine strains. We evaluated cross-reactive antibody responses against two representative A(H3N2) viruses that had antigenically drifted from the A(H3N2) vaccine component using microneutralization (MN) assays. Postvaccination antibody titers to drifted A(H3N2) viruses were higher following receipt of IIV (MN geometric mean titers [GMTs], 63 to 68; 38 to 45% achieved seroconversion) versus LAIV (MN GMT, 22; only 3 to 5% achieved seroconversion). In 9- to 17-year-olds, the highest MN titers were observed among IIV-vaccinated individuals who had received LAIV in the previous season. Among all IIV recipients aged 3 to 17 years, the strongest predictor of antibody responses to the drifted viruses was the prevaccination titers to the vaccine strain. The results of our study suggest that in an antigenically drifted influenza season, vaccination still induced cross-reactive antibody responses to drifted circulating A(H3N2) viruses, although higher antibody titers may be required for protection. Antibody responses to drifted A(H3N2) viruses following vaccination were influenced by multiple factors, including vaccine type and preexisting immunity from prior exposure.

## INTRODUCTION

Neutralizing antibodies against hemagglutinin (HA) on the surfaces of influenza viruses have been considered the major immune mechanism that provides protection against influenza infection (1, 2). However, influenza viruses continuously acquire new mutations on the HA protein through antigenic drift, allowing new variants to escape host immunity. Thus, seasonal influenza vaccines must be updated regularly based on the genetic and antigenic characteristics of the surface HA proteins of circulating viruses (3–5). When hemagglutinins change through antigenic drift, the degree of protection provided by vaccines may be determined by the level of cross-reactive antibodies, although the role of vaccines at providing cross-protection is poorly understood (6, 7). To date, few studies have examined cross-reactive neutralizing antibody responses to antigenically drifted viruses and the implications in vaccine effectiveness (VE).

Among all seasonal influenza virus subtypes, HA of influenza A(H3N2) has the fastest evolutionary rate with new antigenic clusters emerging on average every 3.3 years (8, 9). In a recent meta-analysis, influenza vaccines had reduced effectiveness against illnesses caused by A(H3N2) viruses compared with other influenza virus subtypes (7). In the 2014-2015 influenza season, new clusters of A(H3N2) viruses became predominant (10–13) and were characterized into two genetic groups based on HA sequences: 3C.2a and 3C.3a (14, 15). Viruses in these two genetic groups are antigenically distant from A(H3N2) vaccine strain A/Texas/50/2012 (3C.1) (16), causing antigenic mismatch between the vaccine strain and circulating A(H3N2) viruses. In the United States, estimates of VE against medically attended influenza in the 2014-2015 influenza season were low (17, 18), with a majority of illness caused by A(H3N2) viruses belonging to genetic group 3C.2a (6).

Even when seasonal influenza vaccines are antigenically mismatched to circulating influenza viruses, vaccination may still provide partial protection by inducing cross-reactive antibody responses to circulating strains through shared epitopes on HA or other viral proteins (19). The level of cross-reactivity mainly depends on the genetic and antigenic distance between the vaccine antigen and circulating viruses. Traditionally, antigenic distance between viruses is determined using reference antisera from immunologically naive ferrets infected with influenza viruses. However, in humans, cross-reactive antibodies are also influenced by other factors, including prior immune priming history through influenza infection or vaccination, age, and immune status. Heterologous protection against antigenically drifted strains may also differ between live-attenuated influenza vaccine (LAIV) and inactivated influenza vaccine (IIV) (20, 21).

Here, we investigated immune responses of children and adolescents enrolled in an observational study. We measured serum antibody responses to 2014-2015 live-attenuated and inactivated influenza vaccines, evaluated the levels of neutralizing antibodies to antigenically drifted influenza A(H3N2) strains, and explored factors that may influence cross-reactive antibody responses to drifted A(H3N2) viruses following vaccination.

## MATERIALS AND METHODS

### Study design and setting.

Healthy children aged 3 to 17 years were recruited from three health centers (one pediatric health center and two family medicine health centers) from the University of Pittsburgh Medical Center (UPMC) Health System in 2014. The criteria used for enrollment in the study were as follows: (i) the child had not received and was planning to receive 2014-2015 influenza vaccine; (ii) no contraindications for LAIV; (iii) known vaccination status for the 2013-2014 influenza season (i.e., influenza vaccination documented in medical record or state immunization registry or parent report of no influenza vaccination for the 2013-2014 season); and (iv) weight of ≥17 kg. Due to potential concerns about LAIV safety in those with uncontrolled asthma, children with a history of severe asthma episodes were excluded.

Eligible children were recruited by two age groups, 3 to 8 years and 9 to 17 years. All children received 2014-2015 influenza vaccines either LAIV or IIV according to parent, child, or clinician preference. Children receiving 2014-2015 LAIV were enrolled regardless of prior season vaccination status. Children receiving 2014-2015 IIV were enrolled as the comparison group. All participants were enrolled and vaccinated between September and December 2014. Sera were collected prior to vaccine administration (day 0) and 19 to 33 days postvaccination (mean, 21 days; Table 1). No children were enrolled who required two doses of vaccines. This study was approved by both University of Pittsburgh and Centers for Disease Control and Prevention Institutional Review Boards, and informed written consent was obtained from the parents.

**TABLE 1:** Characteristics of study participants enrolled in the study

Characteristic	No. of study participants (%) or parameter value^*a*^	
	LAIV (*n* = 97)	IIV (*n* = 53)
Age, yr [median (range)]	10 (3–17)	11 (3–17)
Gender		
Female	48 (59)	34 (41)
Male	49 (72)	19 (28)
Age category and 2013-2014 vaccine received		
3- to 8-yr-old	40 (67)^*b*^	20 (33)
IIV	10	18
LAIV	8	0
No vaccine	21	2
9- to 17-yr-old	57 (63)	33 (37)
IIV	16	25
LAIV	12	5
No vaccine	29	3
Paired serum sample collection interval, no. of days [median (range)]	21 (19–33)	21 (20–29)

a The number of study participants or parameter value for study participants who received LAI or IIV vaccine in the 2014-2015 influenza season is given.

b The prior season vaccine type was not determined for one participant in the 3- to 8-year-old LAIV group.

### Hemagglutination inhibition assay.

Hemagglutination inhibition (HI) assays were performed with pre- and postvaccination serum specimens as previously described (22) using 0.5% turkey erythrocytes. Serum samples were treated with receptor-destroying enzyme to remove nonspecific inhibitors. Nonspecific agglutinins were removed by serum adsorption with packed turkey erythrocytes. Serial twofold dilutions of sera were made from an initial 1:10 dilution. The HI titer was defined as the reciprocal of the last dilution of serum that completely inhibited hemagglutination.

Viruses that represent Northern Hemisphere 2014-2015 influenza season quadrivalent vaccine components were used in HI assays: A/California/7/2009 A(H1N1), A/Texas/50/2012 A(H3N2), B/Massachusetts/02/2012 (B/Yamagata lineage), and B/Brisbane/60/2008 (B/Victoria lineage). All viruses used in HI assays were propagated in 9- to 11-day-old embryonic chicken eggs. B virus antigens were ether treated prior to the HI assay.

### Microneutralization assay.

Microneutralization (MN) assays were performed as previously described (22), except the assays were conducted with Madin-Darby canine kidney-SIAT 1 (MDCK-SIAT1) cells. Serum samples were first heat inactivated, and then serial twofold dilutions were made starting at an initial 1:10 dilution. Influenza viruses (100 50% tissue culture infectious doses [TCID_50_]) were added to serum dilutions, incubated at 37°C with 5% CO_2_ for 1 h, and used to infect 1.5 × 10^5^ MDCK-SIAT1 cells per ml. After overnight incubation, the presence of the viral proteins was detected by an enzyme-linked immunosorbent assay (ELISA) using monoclonal antibodies specific to the influenza A virus nucleoprotein. MN titers were defined as the reciprocal of the highest dilution of serum that yielded at least 50% neutralization.

Pre- and postvaccination sera were tested against three A(H3N2) viruses: A/Texas/50/2012 (genetic group 3C.1, 2014-2015 Northern Hemisphere vaccine virus), A/Switzerland/9715293/2013 (group 3C.3a), and A/Nebraska/04/2014 (group 3C.2a). All viruses used in MN assays were propagated in MDCK-SIAT1 cells. Sequence analyses of the viruses were performed using BioEdit alignment editor (http://www.mbio.ncsu.edu/bioedit/bioedit.html).

### Statistical analyses.

For statistical analyses, specimens with reciprocal HI or MN titers of <10 were assigned a titer of 5. Geometric mean titers (GMTs), fold rises or increases (i.e., GMT ratios), and 95% confidence intervals (95% CIs) were calculated using repeated-measure linear mixed models as previously described (23). Seroconversion was defined as a fourfold rise in antibody titers with postvaccination titers of ≥40. Fold rise was calculated as the ratio of the postvaccination titer to the prevaccination titer. HI and MN titers were log_2_ transformed to examine correlations. Linear regression with log_2_-transformed titers was used to examine associations between prevaccination, postvaccination, or fold rise in titer with age category and the prior season (2013-2014) vaccination status. Separate linear regression models restricted to IIV-vaccinated subjects examined predictors of IIV response, including prior season vaccine type. Predictors of seroconversion were examined by logistic regression. Statistical analyses were conducted using SAS for Windows (version 9.3, Cary, NC).

## RESULTS

### Participant characteristics.

Among 150 enrollees aged 3 to 17 years, 97 received quadrivalent LAIV (median age, 10 years) and 53 received quadrivalent IIV (median age, 11 years) in the 2014-2015 influenza season (Table 1). There was no statistical difference in the characteristics of those who received LAIV versus those who received IIV when comparing age, sex, and the interval between paired serum sample collections (*P* > 0.05). At enrollment in the study, the majority of subjects had preexisting HI antibodies to vaccine viruses: HI GMT antibody titers among both IIV and LAIV recipients were 60 to influenza A(H1N1), 119 to A(H3N2), 72 to B/Yamagata, and 35 to B/Victoria vaccine components and were not statistically different between IIV and LAIV recipients (Table 2). Preexisting MN GMT antibody titers to drifted A(H3N2) genetic group 3C.2a and 3C.3a viruses ranged from 18 to 19 and were similar for children who received either IIV or LAIV (Table 3).

**TABLE 2:** HI antibody responses to 2014-2015 vaccine viruses in 3- to 17-year-old LAIV and IIV recipients

Influenza virus strain and prevaccine HI titer^*a*^	2014-2015 LAIV (*n* = 97)							2014-2015 IIV (*n* = 53)						
	No. of subjects (%)	Geometric mean titer (95% CI)		% subjects with the following postvaccine HI titer:		Fold rise	% Seroconversion	No. of subjects (%)	Geometric mean titer (95% CI)		% subjects with with the following postvaccine HI titer:		Fold rise	% Seroconversion
		Prevaccine	Postvaccine	≥40	≥110				Prevaccine	Postvaccine	≥40	≥110		
A/California/07/2009 (A/H1N1)														
All	97 (100)	54 (41–71)	58 (45–75)	69	37	1.1	3	53 (100)	74 (51–106)	246 (189–322)	98	87	3.4	42
HI < 40	31 (32)	10 (8–13)	13 (10–16)	6	0	1.3	6	13 (25)	10 (7–15)	160 (65–394)	92	62	16.0	100
HI ≥ 40	66 (68)	119 (100–142)	119 (100–142)	98	55	1.0	2	40 (75)	141 (114–174)	283 (227–354)	100	95	2.0	23
A/Texas/50/2012 (A/H3N2)														
All	97 (100)	120 (96–151)	131 (103–164)	88	65	1.1	3	53 (100)	118 (86–162)	316 (250–400)	98	91	2.7	34
HI < 40	14 (14)	16 (12–22)	20 (15–26)	84	52	1.2	0	6 (11)	13 (7–26)	151 (38–605)	92	77	11.3	83
HI ≥ 40	83 (86)	168 (140–202)	180 (149–218)	89	71	1.1	4	47 (89)	155 (120–201)	347 (279–432)	100	95	2.2	28
B/Massachusetts/02/2012 (B/Yamagata)														
All	97 (100)	61 (46–81)	101 (81–125)	87	54	1.6	18	53 (100)	98 (73–132)	304 (243–380)	100	92	3.1	34
HI < 40	29 (30)	10 (8–13)	36 (24–55)	74	35	3.6	45	8 (15)	16 (9–28)	247 (114–533)	100	100	15.3	88
HI ≥ 40	68 (70)	132 (108–161)	155 (129–186)	92	62	1.2	6	45 (85)	135 (106–171)	315 (248–401)	100	90	2.3	24
B/Brisbane/60/2008 (B/Victoria)														
All	97 (100)	34 (27–44)	53 (43–65)	73	27	1.5	12	53 (100)	36 (26–49)	148 (115–190)	98	64	4.1	53
HI < 40	40 (41)	0 (8–12)	25 (18–34)	52	19	2.4	25	21 (40)	10 (8–13)	96 (60–153)	92	62	9.3	90
HI ≥ 40	57 (59)	81 (67–97)	90 (75–108)	83	30	1.1	4	32 (60)	83 (68–100)	197 (153–252)	100	65	2.4	28

a The influenza virus strain and prevaccine HI titer are shown for all vaccine recipients and for recipients with HI titers of <40 and ≥40.

**TABLE 3:** Microneutralization antibody responses to A(H3N2) vaccine and drifted viruses among 2014-2015 IIV and LAIV recipients

Influenza virus strain and age group^*a*^	2014-2015 LAIV (*n* = 97)^*b*^				2014-2015 IIV (*n* = 53) ^*b*^			
	Geometric mean titer (95% CI)		Fold rise	% Seroconversion	Geometric mean titer (95% CI)		Fold rise	% Seroconversion
	Prevaccine	Postvaccine			Prevaccine	Postvaccine		
A/Texas/50/2012								
All	178 (135–234)	211 (157–284)	1.2	4	170 (116–247)	565 (418–764)	3.3	42
3- to 8-yr-old	122 (74–200)	133 (81–217)	1.1	0	110 (49–247)	711 (365–1382)	6.5	55
9- to 17-yr-old	232 (170–317)	292 (205–417)	1.3	7	221 (154–317)	491 (366–660)	2.2	33
A/Nebraska/04/2014								
All	18 (15–23)	22 (17–28)	1.2	3	19 (14–25)	63 (45–88)	3.4	38
3- to 8-yr-old	19 (13–28)	20 (17–30)	1.1	0	17 (10–31)	91 (48–175)	5.2	50
9- to 17-yr-old	18 (14–23)	22 (16–31)	1.2	5	19 (14–27)	51 (35–73)	2.6	30
A/Switzerland/9715293/2013								
All	19 (15–24)	22 (17–29)	1.1	5	19 (13–26)	68 (48–96)	3.7	45
3- to 8-yr-old	18 (12–26)	17 (12–26)	1.0	0	15 (9–26)	86 (42–173)	5.8	55
9- to 17-yr-old	21 (15–28)	26 (18–39)	1.3	9	21 (14–32)	59 (40–87)	2.8	39

a The influenza virus strain and age group are shown for all vaccine recipients and for the two age groups.

b LAIV versus IIV postvaccination GMT, fold rise, and seroconversion, *P* < 0.001.

### Antibody responses to influenza A(H1N1), A(H3N2), and B vaccine components.

**FIG 1:**
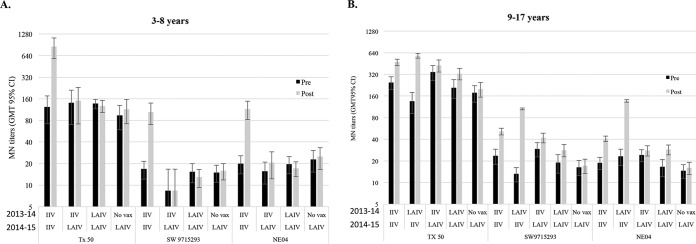
Microneutralization (MN) titers pre- and postvaccination in the 2014-2015 influenza season stratified by age groups and prior season vaccination history. Pre- and postvaccination MN titers were graphed as geometric mean titers (GMTs) with 95% confidence intervals (95% CI). MN antibody responses were stratified by age groups 3- to 8-year-olds (A) and 9- to 17-year-olds (B) and vaccination history from the 2013-2014 and 2014-2015 seasons. MN titer distributions to vaccine virus A/Texas/50/2012 (Tx50) and drifted A(H3N2) viruses A/Switzerland/9715293/2013 (SW9715293), A/Nebraska/04/2014 (NE04) following 2014-2015 IIV and LAIV are shown. No vax, no vaccination.

As measured by HI antibody titers, children who received IIV had higher postvaccination GMTs, seroconversion rates, and fold rises to all four vaccine viruses than those vaccinated with LAIV (*P* < 0.01 for all three comparisons; Table 2). IIV also induced significantly higher MN titers to A(H3N2) vaccine virus than LAIV did (*P* < 0.001; Table 3 and Fig. 1). Overall, 42% of all IIV recipients seroconverted to the A(H3N2) vaccine strain in MN titers, versus only 4% of LAIV recipients who seroconverted (Table 3).

Antibody titers to the influenza A(H3N2) vaccine component measured by HI assay with egg-propagated viruses and by MN assay with MDCK-SIAT1 cell-propagated viruses were highly correlated (Pearson correlation coefficient *r* = 0.90; *P* < 0.001), with reciprocal MN titers being slightly higher (i.e., more sensitive) than the corresponding HI titers: HI titers against A/Texas/50/2012 of 1:40 corresponded to MN titers of ∼1:54.

Among IIV recipients, seroconversion rates and fold rises in HI titers were higher among subjects with lower prevaccination titers (<1:40) than in those with high preexisting titers (≥1:40) for all four vaccine viruses (*P* < 0.01; Table 2). LAIV recipients had low seroconversion rates and fold rises in HI titers to influenza A(H3N2) and A(H1N1) vaccine viruses regardless of prevaccination HI titers, but seroconversion rates were higher against the B/Yamagata and B/Victoria vaccine viruses among LAIV recipients with low prevaccination titers (<40) than those with high prevaccination titers (≥40) (*P* < 0.05; Table 2).

### Cross-reactive neutralizing antibodies to antigenically mismatched circulating influenza A(H3N2) viruses.

Compared to the A(H3N2) vaccine strain A/Texas/50/2012, A/Nebraska/04/2014 (genetic group 3C.2a) and A/Switzerland/9715293/2013 (group 3C.3a) viruses have mutations in several positions in HA proteins, including antigenic sites A and B, and near the receptor binding pocket (see Table S1 in the supplemental material); both are antigenically distinct from the A(H3N2) vaccine strain characterized with ≥8-fold reduction in HI titers when tested with ferret antisera against the vaccine strain (data not shown).

Vaccination with influenza A/Texas/50/2012 induced cross-reactive neutralizing antibodies to drifted A(H3N2) viruses from genetic groups 3C.2a and 3C.3a, with MN titers against drifted viruses 7- to 11-fold lower than against vaccine virus (Table 3). Among IIV recipients, neutralizing antibody titers against drifted A(H3N2) viruses increased proportionally to response to vaccine virus with fold rises in MN titers ranging from 3.3 to 3.7 (Table 3). When the subjects were stratified by age, increases in MN titers against A(H3N2) vaccine as well as drifted viruses were higher among IIV recipients aged 3 to 8 years, who also had lower prevaccination titers, compared to subjects aged 9 to 17 years (Table 3).

**FIG 2:**
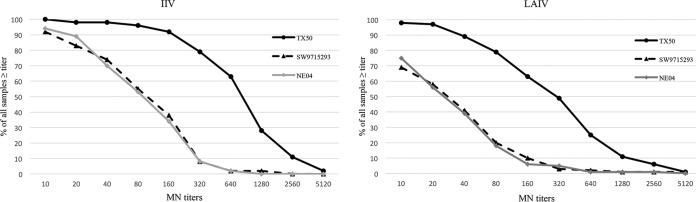
Microneutralization (MN) titer distributions to A(H3N2) vaccine and drifted viruses postvaccination with 2014-2015 IIV and LAIV. MN titer distributions to vaccine virus A/Texas/50/2012 (TX50) and drifted A(H3N2) viruses A/Switzerland/9715293/2013 (SW9715293), A/Nebraska/04/2014 (NE04) following 2014-2015 IIV and LAIV are shown.

Among IIV recipients, ≥70% and ≥40% had postvaccination MN titers of ≥40 and ≥110 against drifted influenza A(H3N2) viruses, respectively, although postvaccination GMTs reached only 63 (95% CI, 45 to 88) for A/Nebraska/04/2014 (group 3C.2a) and 68 (95% CI, 48 to 96) for A/Switzerland/9715293/2013 (group 3C.3a) compared to 565 (95% CI, 418 to 764) for the vaccine virus. Among LAIV recipients, approximately 40% and 10% had postvaccination MN titers of ≥40 and ≥110 against the drifted H3N2 vaccine virus, respectively, with MN GMT around 22 postvaccination; pre- and postvaccination GMTs were not significantly different (Fig. 2 and Table 3).

### Factors associated with cross-reactive neutralizing antibody responses to drifted influenza A(H3N2) viruses.

Receipt of LAIV in the 2014-2015 influenza season was not associated with increased MN titers against drifted A(H3N2) viruses, even after controlling for age group, prevaccination MN titer, and prior season vaccine type (Table 3). However, among 48 children vaccinated in the 2013-2014 influenza season who received IIV in the 2014-2015 season, the largest increase in neutralizing antibody responses to drifted A(H3N2) viruses following IIV receipt was observed in children who received LAIV in the prior season (all in the 9- to 17-year age group; Table 4). Among 9 to 17-year-olds, in a linear regression model controlling for prevaccination MN titers, postvaccination MN titers to drifted A(H3N2) viruses were higher (*P* = 0.002 for A/Nebraska/04/2014; *P* = 0.07 for A/Switzerland/9715293/2013) in children vaccinated with LAIV in the 2013-2014 season than in those vaccinated with IIV in the prior season. Among all children vaccinated with IIV in the 2014-2015 season, prevaccination MN titers to the A(H3N2) vaccine strain were the strongest predictor of seroconversion to drifted 3C.2a and 3C.3a viruses (*P* < 0.05), with higher likelihood of seroconversion at lower prevaccination titers against the A(H3N2) vaccine virus (see Table S2 in the supplemental material).

**TABLE 4:** Microneutralization antibody titers to A(H3N2) viruses following 2014-2015 IIV vaccination (*n* = 48) stratified by age and prior season vaccine type^*a*^

Influenza virus strain and age group	2013-2014 vaccination	No. of subjects	Geometric mean titer (95% CI)		% subjects with the following postvaccine MN titer:		Fold rise	% Seroconversion
			Prevaccine	Postvaccine	≥40	≥110		
A/Texas/50/2012 (3C.1)								
3- to 8-yr-old	IIV	18	124 (51–300)	855 (434–1,686)	94	94	6.9	61
9- to 17-yr-old	IIV	25	269 (175–413)	478 (354–646)	100	100	1.8	20
	LAIV	5	150 (53–426)	926 (353–2,430)	100	100	6.2	100
A/Nebraska/04/2014 (3C.2a)								
3- to 8-yr-old	IIV	18	20 (11–36)	115 (63–212)	89	72	5.8	50
9- to 17-yr-old	IIV	25	20 (13–30)	40 (27–60)	56	20	2.0	8
	LAIV	5	25 (11–57)	176 (81–379)	100	80	7.1	80
A/Switzerland/9715293/2013 (3C.3a)								
3- to 8-yr-old	IIV	18	17 (9–30)	105 (52–211)	83	72	6.2	50
9- to 17-yr-old	IIV	25	24 (15–41)	51 (33–79)	68	32	2.1	20
	LAIV	5	13 (6–28)	124 (26–599)	80	60	9.4	80

a Note that five study participants who were not vaccinated in the 2013–2014 season were excluded from this analysis.

## DISCUSSION

Despite the poor antigenic match between the 2014-2015 influenza A(H3N2) vaccine component and circulating influenza A(H3N2) viruses, children and adolescents vaccinated with inactivated influenza vaccine exhibited significant antibody rises and seroconversion to A(H3N2) vaccine virus as well as to newly emerged, antigenically drifted influenza A(H3N2) viruses that circulated during the 2014-2015 influenza season. Overall, more than 70% of IIV-vaccinated children had MN titers of ≥40 after vaccination and 38 to 45% seroconverted to A(H3N2) vaccine and drifted viruses. However, postvaccination GMTs for cross-reactive neutralizing antibodies to drifted A(H3N2) viruses remained almost 10-fold lower than antibodies against the vaccine component, with geometric mean MN titers ranging from 63 to 68 to drifted A(H3N2) viruses. In contrast to IIV, receipt of LAIV did not significantly increase titers to vaccine or cross-reactive neutralizing antibodies to drifted viruses. Data from the U.S. Influenza Vaccine Effectiveness network reported low VE in children and adolescents for this season: only −5% (95% CI, −40% to 21%) VE for LAIV and 13% (95% CI, −9% to 30%) VE for IIV against influenza A(H3N2)-related illness (18). Thus, MN titers of ≥40 and seroconversion alone likely did not predict cross-protection against drifted A(H3N2) viruses.

Immune correlates of protection are not well established among children and may differ between inactivated and live attenuated vaccines. Traditionally, a postvaccination HI titer of 40 has been associated with 50% reduction in influenza infection in adults (24–27). The MN titer threshold that correlates with protection is less well defined. In a household study, a MN titer of 40 was demonstrated to be correlated with 49% protection against PCR-confirmed influenza A(H3N2) infections. In the same study, HI titer of 40 was associated with only 31% protection against A(H1N1) and A(H3N2) (28). This lower protection estimate from HI titer of 40 (suggesting that a higher threshold is required for 50% protection) is likely due to the household study setting with exposures of greater duration and intensity. The protective threshold can also vary with age. A higher threshold of HI titer at 110 has been associated with providing 50% protection against A(H3N2) in young children (29). Higher antibody levels needed in children could be due to lower levels of cellular immunity and lack of prior immunity to influenza viruses through prior vaccination and/or infection. In a more recent study that used MN assays in children and adolescents aged 3 to 15 years of age, MN titers as high as 320 were associated with 60% protection in a season during which vaccine and circulating A(H3N2) viruses were well matched (30).

In this study, MN assays were used to evaluate cross-reactivity of antibodies to circulating influenza A(H3N2) viruses. Traditional HI assays are not possible for many A(H3N2) viruses belonging to genetic group 3C.2a, due to their altered receptor binding properties to red blood cells, resulting in insufficient hemagglutination activity for characterization by HI (16, 31). Few studies have used MN assays to measure cross-reactive antibodies to antigenically drifted influenza viruses, in part due to the greater technical complexity of the assay. The MN assay directly measures the ability of antibodies to neutralize influenza virus replication in mammalian cell culture *in vitro*, whereas the HI assay is considered only a surrogate assay. Our data and several previous studies demonstrated higher sensitivities for the MN assay than for the HI assay (30, 32). Children and adolescents enrolled in this study had a high level of preexisting neutralizing antibodies to vaccine viruses, and many, especially those vaccinated with IIV, reached postvaccination MN titers of ≥40 and ≥110 against drifted A(H3N2) viruses. Approximately 45% of IIV recipients and 10% of LAIV recipients achieved MN titers of ≥110 to drifted A(H3N2) variants. These do not appear to be associated with the level of VE observed in this season. If these levels of MN antibodies are associated with protection, we would have expected higher estimates of IIV vaccine effectiveness against the predominant drifted A(H3N2) viruses than those reported during the 2014-2015 season (18). It is also worth noting that in current VE estimates (18), the incidence of medically attended influenza in vaccinated groups was compared with those in unvaccinated groups. When unvaccinated individuals have preexisting baseline titers to drifted strains due to cross-reactivity from past influenza vaccination or infection, the antibody levels required in the vaccinated group to achieve meaningful VE may be higher. Nevertheless, the preexisting antibodies to drifted A(H3N2) strains at baseline in these children were low (MN GMT of <20). Taken together, our data suggest that heterologous protection in children and adolescents against drifted A(H3N2) viruses during the 2014-2015 influenza season may have required higher levels of cross-reactive antibodies than observed in this study.

We examined factors that may contribute to levels of cross-protective antibodies to drifted viruses postvaccination. An individual's antibody repertoire accumulated from past priming history to influenza viruses can shape the responses to current influenza vaccination (33, 34). Preexisting antibody titers to the influenza A(H3N2) vaccine component were correlated with cross-reactive neutralizing antibodies to the drifted A(H3N2) viruses prior to vaccination. Prevaccination titers to the A(H3N2) vaccine strain, rather than prior season vaccination type and age, was the strongest predictor of seroconversion to drifted A(H3N2) 3C.2a and 3C.3a viruses, although we were unable to disentangle the association between prevaccination titers with age and prior season vaccination. Influenza vaccine can stimulate expansion of memory B cells that produce antibodies recognizing shared epitopes between the vaccine virus and antigenically related viruses the individual may have been exposed to earlier in life. The complexity of an individual's antibody landscape often increases with age (33, 35, 36). Even with fairly young age groups enrolled in this study, we observed higher baseline titers in older children (aged 9 to 17 years) than in younger children. In addition, prior encounter with wild-type influenza virus (infection) or attenuated live virus through LAIV vaccination may stimulate cell-mediated immunity that can later aid the cross-reactive humoral responses when boosted with IIV (37–40). Among 9- to 17-year-olds vaccinated with IIV in the 2014-2015 season, receipt of LAIV in the prior season was associated with higher cross-reactive antibody responses to drifted A(H3N2) viruses than receipt of IIV in the prior season. Further studies are needed to elucidate complex host factors that will shape cross-protective humoral responses after influenza vaccination.

Consistent with low vaccine effectiveness observed for LAIV against influenza A(H3N2)-related illness during the 2014-2015 influenza season (18), receipt of LAIV resulted in limited antibody responses to the A(H3N2) vaccine component A/Texas/50/2012 and little increase in cross-reactive neutralizing antibodies to drifted 3C.2a and 3C.3a A(H3N2) viruses. Evidence of reduced LAIV effectiveness compared to IIV against A(H1N1)pdm09 viruses since 2013 (41, 42), together with the failure of LAIV to provide protection against drifted A(H3N2) viruses in the 2014-2015 season (18), led the Advisory Committee on Immunization Practices to vote in June 2016 that LAIV should not be used in the 2016-2017 season (43). One of the challenges for the use of LAIV is the lack of clear immune correlates of protection (44). For IIV, serum antibody responses after vaccination is a well-established immune marker to assess vaccine immunogenicity, whereas LAIV is thought to offer immune protection mainly through mucosal and cell-mediated immunity. Other immune markers, such as mucosal IgA and cell-mediated responses have been hypothesized to be better correlates of protection for LAIV (45). However, in a recent study, these immune markers were not associated with protection against a LAIV challenge (44). Further, the mechanism of LAIV protection against antigenically drifted strains is not well understood. In one randomized trial, LAIV provided superior protection than IIV against H3N2 viruses that differed antigenically from vaccine virus (46). In other studies, IIV was more efficacious than LAIV in preventing influenza infection to drifted A(H3N2) viruses (20, 21). In addition, preexisting antibodies are thought to interfere with immune response to LAIV (45). However, in our study, poor response to LAIV A(H1N1) and A(H3N2) vaccine viruses was observed even among subjects with low prevaccination titers. In comparison, antibody titers to influenza B viruses in LAIV increased significantly among children with low prevaccination titers, suggesting that antibody responses following LAIV may differ by vaccine component.

Our study has several limitations. Surveillance was not conducted for influenza illnesses among study participants, so we cannot correlate antibody levels with actual protection. Enrollees were not randomized by vaccine type, and there may be differences between LAIV and IIV recipients that affect immune responses, although when comparing characteristics such as age, sex, and preexisting titers to vaccine antigens, there was no statistical difference between LAIV- and IIV-vaccinated children. In addition, vaccination history was determined for only one prior season (2013-2014 season). We do not have vaccination information from earlier seasons and also lack information on possible infections. The absence of this information may contribute to the lack of association between 2013-2014 vaccination history and prevaccination titers. Prior infection and repeated vaccination may change the antibody profile and affect the cross-reactive antibody responses to the antigenically mismatched circulating strains. Also, we were able to enroll only a small number of children previously vaccinated with LAIV or unvaccinated children who chose to receive IIV in the 2014-2015 season. When stratified by both age categories and prior season vaccination, sample sizes in strata were small.

In summary, IIV induced cross-reactive neutralizing antibodies to drifted A(H3N2) viruses that predominated during the 2014-2015 influenza season in children, but higher thresholds may be required for heterologous protection. Further research is needed to better understand the correlate of protection against antigenically drifted viruses, and ultimately to develop improved vaccines and vaccination strategies that can offer broader immune protection to overcome the challenges from constant antigenic drift of seasonal influenza viruses.

## Supplementary Material

Supplemental material
