# Supplementary material for: Neutralizing Antibody Responses to Antigenically Drifted Influenza A(H3N2) Viruses among Children and Adolescents following 2014-2015 Inactivated and Live Attenuated Influenza Vaccination
Source: Clin Vaccine Immunol. 2016 Oct 4;23(10):831–9. doi: 10.1128/CVI.00297-16 (PMC5051070; doi:10.1128/CVI.00297-16)
Supplement: Supplemental material [file supp_23_10_831__index.html]

Supplemental material 

# Neutralizing Antibody Responses to Antigenically Drifted Influenza A(H3N2) Viruses among Children and Adolescents following 2014-2015 Inactivated and Live Attenuated Influenza Vaccination

## Supplemental material

**Files in this Data Supplement:**

- Supplemental file 1 -

  Table S1. Genetic features of HA proteins of A(H3N2) vaccine strain (3C.1) and 3C.3a and 3C.2a strains used in the study. Table S2. Predictors of neutralizing antibody responses (i.e., seroconversion) to A(H3N2) viruses for children and adolescents who received IIV in 2014?2015.

  PDF, 42K
