# Supplementary material for: Neutralizing Antibody Responses to Antigenically Drifted Influenza A(H3N2) Viruses among Children and Adolescents following 2014-2015 Inactivated and Live Attenuated Influenza Vaccination
Source: Clin Vaccine Immunol. 2016 Oct 4;23(10):831–9. doi: 10.1128/CVI.00297-16 (PMC5051070; doi:10.1128/CVI.00297-16)
Supplement: Supplemental material [file CVI.00297-16_zcd999095405so1.pdf]

Supplementary table 1. Genetic features of HA proteins of A(H3N2) vaccine strain (3C.1), and 3C.3a, 3C.2a strains used in the study. Amino acid (AA) differences of 3C.2a and 3C.3a viruses as compared to vaccine strain (3C.1).

HA (AA #)	Antigenic site	A/Texas/50/2012[a] (3C.1)	A/Switzerland/9715293/2013[a] (3C.3a)	A/Nebraska/04/2014[a] (3C.2a)
3		L		I
94		Y		H
128	B	N	A	T
138	A	A	S	
142	A	R	G	
144	A	N		S
145	A	N	S	S
159	B	F	S	Y
160	B	K		T
198	B	P	S	S
225	RBS[b]	N	D	D
311	C	Q		H
326		K	R	
489		D		N

a Accession numbers: A/Texas/50/2012: Genbank KC892952, A/Switzerland/ 9715293/2013: GISAID EPI541659, A/Nebraska/04/2014 EPI537000.

b Receptor binding site

Supplementary table 2: Predictors of neutralizing antibody responses (i.e. seroconversion) to A(H3N2) viruses for children and adolescents who received IIV in 2014–2015

Predictors	A/Texas/50/2012 Parameter estimate (SE[a])	p-value	A/Switzerland/9715293/2013 Parameter estimate (SE[a])	p-value	A/Nebraska/04/2014 Parameter estimate (SE[a])	p-value
**Age group**						
3–8 years	REF		REF		REF	
9–17 years	-0.8 (0.7)	0.3	-0.5 (0.7)	0.4	-0.6(0.7)	0.4
**2013–2014 vaccine received**						
LAIV/No vaccine	REF		REF		REF	
IIV	-0.8 (0.8)	0.3	-1.1 (0.8)	0.2	-0.8 (0.9)	0.3
**Pre-vaccination MN titer to**						
A/Texas/50/2012	-0.4 (0.2)	0.03 [b]	-0.4 (0.2)	0.03 [b]	-0.6 (0.2)	0.005 [b]

[a] SE: Standard Error

[b] Statistically significant (P<0.05)
